# Widerlegung des Mythos vom „Abszesswetter“, welches das Auftreten von Peritonsillitiden und Peritonsillarabszessen begünstigt

**DOI:** 10.1007/s00106-023-01384-w

**Published:** 2023-11-06

**Authors:** Franziska von Meyer, Katharina Storck

**Affiliations:** Klinik und Poliklinik für Hals-Nasen-Ohrenheilkunde, Klinikum rechts der Isar, Technische Universität München, Ismaningerstraße 22, 81675 München, Deutschland

**Keywords:** Eiter, Abszess, EBV Tonsillitis, Tonsillitis, Streptokokken, Pus, Abscess, EBV tonsillitis, Tonsillitis, Streptococcus

## Abstract

**Hintergrund:**

Der Peritonsillarabszess (PTA) wird häufig als Komplikation der akuten Tonsillitis gesehen und ist definiert als Eiterverhalt zwischen der Tonsillenkapsel und dem peritonsillären Gewebe. Die Ätiologie und Pathogenese sind bisher noch nicht vollständig geklärt. Ein Zusammenhang zwischen bestimmten Wetterbedingungen und Temperaturschwankungen und dem Auftreten von Abszessen im Kopf-Hals-Bereich wird seit Jahren diskutiert. Hierbei ist die Frage, ob höhergradige Temperaturschwankungen prädisponierend sind für die Ausbildung von Abszessen.

**Material und Methodik:**

Es erfolgte eine retrospektive Auswertung aller Patienten, die in einem Zeitraum von 10 Jahren (2012–2021) in der Klinik und Poliklinik für Hals, Nasen‑, Ohrenheilkunde des Klinikums rechts der Isar der Technischen Universität München mit einer Peritonsillitis oder einem PTA stationär behandelt wurden. Jeder Patient wurde einzeln mit den täglichen Temperaturdaten des statistischen Wetteramts für die Stadt München korreliert.

**Ergebnisse:**

Insgesamt konnten 1450 Patienten in die Studie eingeschlossen werden. Von den 1450 Patienten hatten 270 Patienten (18,62 %) eine Peritonsillitis, 1180 Patienten (81,38 %) einen PTA. Eine Korrelation zwischen dem Auftreten von Peritonsillitiden oder PTA und größeren Temperaturschwankungen konnte in diesem großen Patientenkollektiv ausgeschlossen werden. Auch zeigte sich über das ganze Jahr eine ähnliche Häufigkeit von Peritonsillitiden und PTA.

**Schlussfolgerung:**

Peritonsillitiden oder Peritonsillarabszesse entwickeln sich nach Datenlage der vorliegenden Studie wetterunabhängig.

## Abstract (en)

**Background:**

Peritonsillar abscess (PTA) is often seen as a complication of acute tonsillitis and is defined as pus retention between the tonsillar capsule and the peritonsillar tissue. The etiology and pathogenesis have not yet been fully elucidated. A connection between certain weather conditions and temperature fluctuations and the occurrence of abscesses in the head and neck region has been discussed for years. The question here is whether higher temperature fluctuations are predisposing for the formation of abscesses.

**Materials and methods:**

A retrospective evaluation of all patients hospitalized with peritonsillitis or PTA in the Department of Otorhinolaryngology of the Klinikum Rechts der Isar of the Technical University of Munich during a period of 10 years (2012–2021) was performed. Each patient was individually correlated with daily temperature data from the statistical meteorological office of the City of Munich.

**Results:**

A total of 1450 patients were included, 270 patients (18.62%) with peritonsillitis and 1180 patients (81.38%) with PTA. A correlation between the occurrence of peritonsillitis or PTA and major temperature fluctuations could be excluded in this large patient population. Moreover, a similar frequency of peritonsillitis and PTA was seen throughout the year.

**Conclusion:**

The myth of a temperature dependence of the development of peritonsillitis or PTA and a so-called abscess weather could be negated in this study.

Fast 5 % aller Beratungsanlässe in der Hals-Nasen-Ohren-ärztlichen oder hausärztlichen Praxis sind bakterielle Tonsillopharyngitiden [3]. Differenzialdiagnostisch kommen virale Pharyngitiden wie eine Infektion mit dem Epstein-Barr-Virus (EBV) infrage. Eine Herausforderung stellt die Differenzierung zwischen einer Tonsillitis, Peritonsillitis und einem Peritonsillarabszess (PTA) als Indikation für eine stationäre Einweisung dar.

Der PTA ist definiert als Eiterverhalt zwischen der Tonsillenkapsel und dem Musculus constrictor pharyngis. Seine Ätiologie und Pathogenese sind nicht vollständig geklärt. Im Allgemeinen gilt der PTA als Komplikation einer akuten Tonsillitis mit dem Zwischenstadium einer Peritonsillitis, andere Mechanismen in der Entstehung, wie eine Obstruktion der supratonsillären Weber-Drüsen, werden ebenfalls diskutiert. [3, 4]. Die Peritonsillitis bezeichnet die Ausbreitung auf das Peritonsillargewebe ohne Eiterverhalt [3, 16]. Typische Symptome des PTA sind Odyno‑/Dysphagie, kloßige Sprache, Kieferklemme und beginnende Dyspnoe [14, 31]. Zumeist zeigt sich eine unilateral geschwollene Tonsille, eine Rötung und Schwellung des Weichgaumens, Asymmetrie des Oropharynx sowie ein Uvulaödem begleitet von Fieber und Abgeschlagenheit [3, 16].

Eine schnelle Diagnostik sowie Therapieeinleitung sind essenziell. Eine unbehandelte Infektion kann zu einer Ruptur des Abszesses mit Eiteraspiration oder einem Absinken des Abszesses mit Obstruktion der Atemwege, Infektion der tiefen Halsweichteile bis hin zu einer nekrotisierenden Fasziitis und Mediastinitis führen [1, 4, 16].

Der Haupterreger der akuten bakteriellen Tonsillitis ist der Streptococcus pyogenes (Gruppe-B-Streptokokken), welcher für 15–30 % der akuten Tonsillitiden im Kindesalter und für 5–10 % der Tonsillitiden im Erwachsenenalter verantwortlich sein soll [3]. Bei PTA besteht meist eine aerob-anaerobe Mischinfektion aus Streptokokken der Gruppe A, Fusobacterium necrophorum, Peptostreptokokken und Prevotella spp. [3, 4, 17].

Die Therapie des PTA besteht in einer antibiotischen Therapie und Abszessdrainage. Diese kann über eine Nadelpunktion, Inzisionsdrainage oder Tonsillektomie erfolgen [3, 14, 31]. Da die Peritonsillitis oft ein Übergangsstadium zum PTA darstellt, ist eine stationäre Aufnahme und intravenöse antibiotische Therapie empfohlen [11]. Eine tägliche Reevaluation ist ratsam, um einen beginnenden Abszess zu erkennen.

Als Risikofaktoren für einen PTA wurden Veränderungen der oropharyngealen Flora, vermehrte Infektionen (z. B. EBV), Rauchen und eine schlechte Mundhygiene beschrieben [16, 20].

Ein Zusammenhang zwischen Klimabedingungen und der Entstehung von Krankheiten wird bereits seit Langem untersucht. Bereits vor ca. 2000 Jahren wurde in der Schrift „Über die Umwelt“ (*De aere aquis locis*), eine der ältesten Schriften des Corpus Hippocraticum, untersucht, ob bestimmte Umweltbedingungen und Jahreszeiten einen Einfluss auf die Entstehung von Entzündungen und Abszesse haben können [21]. Auch zeigte sich bereits in einigen Studien, dass das Auftreten bestimmter entzündlicher Erkrankungen wie Phlegmonen, Appendizitiden oder odontogene Abszesse bei wärmeren Temperaturen erhöht ist [5, 26, 27]. Ein Zusammenhang zwischen bestimmten Wetterbedingungen und dem Auftreten von Kopf-Hals-Abszessen wird ebenfalls seit Längerem vermutet, wurde bisher jedoch nicht wissenschaftlich belegt.

Einige Studien berichten von saisonalen Schwankungen in der Häufigkeit des Auftretens von PTA, während andere keine statistisch relevante Schwankung nachweisen konnten [17, 18]. Bei allen Studien wurde das Wetter/die Temperaturen jedoch nicht individuell mit jedem Patienten korreliert, sondern lediglich die monatliche Durchschnittstemperatur ausgewertet [10, 11, 24, 29].

Ziel dieser retrospektiven Studie war es zu untersuchen, ob hohe Temperaturschwankungen zwischen der minimalen und der maximalen Temperatur (Temperaturrange) in den Tagen vor Auftreten eines Abszesses einen Einfluss auf dessen Entstehung haben. Während kalte Temperaturen Tonsillopharyngitiden begünstigen, wird vermutet, dass Abszesse vor allem bei warmen Temperaturen entstehen. Eine große Range könnte hierbei begünstigend sein. Hierzu wurden die Häufigkeiten von Peritonsillitiden/PTA sowie weitere demografische und krankheitsbezogene Parameter wie Alter, Beschwerdedauer, Krankenhausaufenthaltsdauer, laborchemische Infektparameter, Therapien und Erregerspektrum in einer aufwendigen Statistik individuell patientenbezogen untersucht.

## Methode

Es erfolgte die retrospektive Auswertung von 1450 Patienten, die im Zeitraum von 10 Jahren (2012–2021) in der Klinik und Poliklinik für Hals‑, Nasen‑, Ohrenheilkunde des Klinikums rechts der Isar der Technischen Universität München im Rahmen einer Peritonsillitis/PTA stationär aufgenommen wurden. Die Suchabfrage erfolgte über das SAP System Net Weaver Health Care (Version 7.4, SAP SE, Walldorf, Deutschland). Eingeschlossen wurde der Diagnoseschlüssel J36.0 nach ICD 10 für Peritonsillitis/PTA. Die digitalen Krankenakten enthielt alle Informationen über klinische Symptome, Beschwerdedauer, Alter, Einweisungsdiagnose, Aufenthaltsdauer, Therapie und Infektparameter.

Diese Studie wurde von der Ethikkommission an der Technischen Universität München bewilligt (Bewilligungsnummer 2023-207-S-KH).

Die Abstriche wurden vom Institut für medizinische Mikrobiologie der Technischen Universität München ausgewertet. Die laborchemischen Parameter wurden durch das Institut für klinische Chemie ausgewertet (Datenbank SWISSLAB, Lauris Version 2.21.10).

Die täglichen meteorologischen Daten der Stadt München und Umgebung von 2012–2021 wurden vom Deutschen Wetterdienst bereitgestellt. Diese beinhalteten auch das tägliche Temperaturminimum und -maximum. Um einen Zusammenhang zwischen den täglichen und mehrtägigen Temperaturschwankungen (Temperaturrange) und der Entwicklung von PTA zu eruieren, erfolgte in Zusammenarbeit mit einer externen Firma für medizinische Statistik die komplexe Auswertung der Temperaturdaten für jeden einzelnen Patienten.

Hierbei wurden für jeden Patienten das tägliche Temperaturmaximum und -minimum, der Mittelwert und die maximale Temperaturrange von Beschwerdebeginn bis zur Vorstellung errechnet. Als Zeitintervall für die Auswertung wurden 7 Tage bis zur Vorstellung in der Klinik gewählt.

Die statistische Analyse erfolgte durch eine externe Firma für medizinische Statistik mit der Software SPSS Statistics for Windows unter Verwendung von Chi-Quadrat- und zweiseitigen t‑Tests. T‑Tests für unabhängige Stichproben wurden verwendet, um potenzielle Zusammenhänge zwischen dem Auftreten von Peritonsillitiden und PTA und Klimafaktoren (durchschnittliche Temperatur, minimale und maximale Temperatur) zu bewerten. *p*-Werte von 0,05 oder weniger wurden als signifikant angesehen.

## Ergebnisse

Zwischen 01/2012 und 12/2021 wurden 1450 Patienten mit Peritonsillitis/PTA in unserer Klinik stationär behandelt. Von den 1450 Patienten hatten 270 Patienten (18,62 %) eine Peritonsillitis, 1180 Patienten (81,38 %) einen PTA (Tab. 1).

**Table 1:** 

Variablen	Anzahl *n, *Häufigkeit	Mittelwert	Standardabweichung	50 % (Median)
*Gesamtkollektiv*				
Alter bei Erstvorstellung (Jahre)	1450	36,21	15,97	33,37
Beschwerden seit wann (Tage)	1449	4,69	3,27	4,00
CRP bei Aufnahme (mg/dl)	1450	8,96	7,35	6,80
Leukozyten bei Aufnahme (G/l)	1450	13,74	4,76	13,33
Stationäre Aufnahme (Tage)	1450	5,15	3,01	5,00
*Peritonsillitis*				
Alter bei Erstvorstellung (Jahre)	270	36,26	14,74	34,66
Beschwerden seit wann (Tage)	270	4,69	3,27	4,00
CRP bei Aufnahme (mg/dl)	270	7,83	6,58	5,8
Leukozyten bei Aufnahme (G/l)	270	12,77	4,22	12,78
Stationäre Aufnahme (Tage)	270	3,81	1,62	4,00
*Peritonsillarabszess*				
Alter bei Erstvorstellung (Jahre)	1180	36,20	16,24	33,15
Beschwerden seit wann (Tage)	1180	4,69	3,272	4,00
CRP bei Aufnahme (mg/dl)	1180	9,23	7,50	7,20
Leukozyten bei Aufnahme (G/l)	1180	13,97	4,85	13,54
Stationäre Aufnahme (Tage)	1180	5,46	3,16	5,00

Das mittlere Alter bei Erstvorstellung mit Peritonsillitis lag bei 36,26 (± 14,74) Jahren, bei PTA bei 36,20 (± 16,24) Jahren. Es zeigte sich kein statistisch signifikanter Unterschied zwischen dem Alter bei Erstvorstellung bei Peritonsillitis/PTA (*p* = 0,953).

Die mittlere Beschwerdedauer (u. a. Odynophagie, Dysphagie, beginnende Kieferklemme) bei Peritonsillitis und PTA vor der Vorstellung in unserer HNO-Ambulanz lag im Mittel bei 4,69 (± 3,24) Tagen.

### Temperaturabhängigkeit

Die Durchschnittstemperatur lag bei Patienten mit Peritonsillitis bei 10,2 °C (± 6,89; Minimum 3,3 °C, Maximum 19,9 °C). Die Temperaturrange zwischen niedrigster und höchster Temperatur in der Woche vor Erstvorstellung bei Patienten mit Peritonsillitis bei 8,8 °C (± 2,73; Tab. 2).

**Table 2:** 

Variablen	Anzahl *n, *Häufigkeit	Mittelwert	Standardabweichung	50 % (Median)
*Gesamtkollektiv*				
Temperaturminimum (°C)	1449	3,87	6,78	3,40
Temperaturmaximum (°C)	1449	20,50	8,56	21,00
Temperaturrange, Durchschnitt (°C)	1449	9,02	2,73	9,14
Temperaturdurchschnitt (°C)	1448	19,96	7,23	10,70
*Peritonsillitis*				
Temperaturminimum (°C)	270	3,33	6,27	2,45
Temperaturmaximum (°C)	270	19,86	9,31	19,50
Temperaturrange, Durchschnitt (°C)	270	8,80	2,73	9,00
Temperaturdurchschnitt (°C)	270	10,24	6,89	9,47
*Peritonsillarabszess*				
Temperaturminimum (°C)	1179	3,99	6,88	3,80
Temperaturmaximum (°C)	1179	20,64	8,63	21,30
Temperaturrange, Durchschnitt (°C)	1179	9,07	2,72	9,16
Temperaturdurchschnitt (°C)	1179	11,12	7,30	11,03

**Figure 1:**
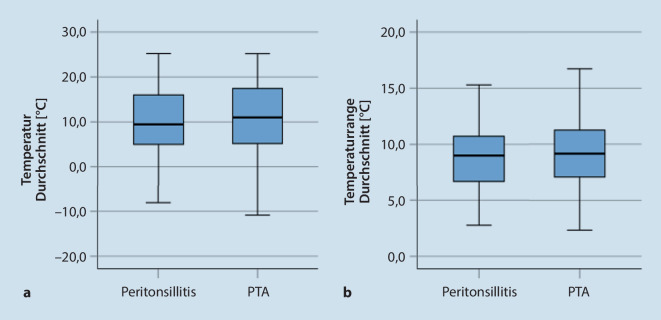

Die Durchschnittstemperatur bei Patienten mit PTA lag bei 11,1 °C (± 7,30). Die Temperaturrange bei 9,1 °C (± 2,72; Abb. 1a, b). Es zeigte sich keine statistisch signifikante Abhängigkeit zwischen der Anzahl an Patienten mit einer Peritonsillitis/PTA und der Durchschnittstemperatur oder Temperaturrange in der Woche vor Erstvorstellung (*p* > 0,05).

**Figure 2:**
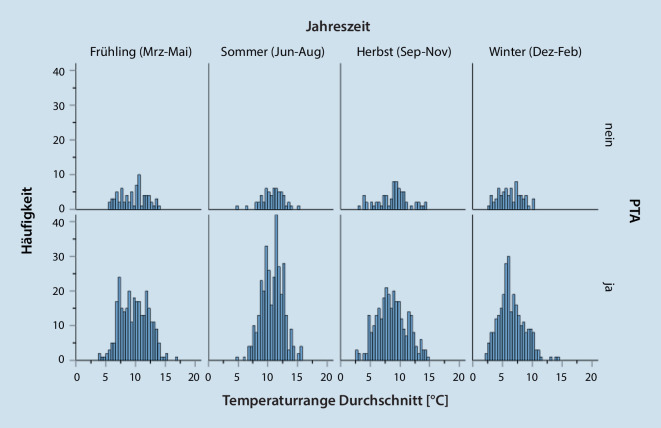

Auf alle Monate sah man eine relative Gleichverteilung für das Auftreten von Peritonsillitiden/PTA. Der Juni zeigte eine leicht erhöhte Häufigkeit ohne statistische Signifikanz (Tab. 3; Abb. 2).

**Table 3:** 

Monat	Peritonsillitis: Anteil in % (Anzahl *n* der Patienten)	PTA: Anteil in % (Anzahl *n* der Patienten)	Gesamt: Anteil in % (Anzahl *n* der Patienten)
Januar	9,3 (25)	8,6 (101)	8,7 (126)
Februar	7,0 (19)	7,5 (88)	7,4 (107)
März	9,6 (26)	8,0 (94)	8,3 (120)
April	10,4 (28)	7,6 (90)	8,1 (118)
Mai	6,7 (18)	8,9 (105)	8,5 (123)
Juni	6,3 (12)	10,3 (122)	9,6 (139)
Juli	8,1 (22)	8,9 (105)	8,8 (127)
August	7,0 (19)	9,1 (107)	8,7 (126)
September	6,8 (18)	7,4 (87)	7,2 (105)
Oktober	11,1 (30)	9,1 (107)	9,4 (137)
November	8,1 (22)	8,1 (95)	8,1 (117)
Dezember	9,6 (26)	6,7 (79)	7,2 (105)

Auch die Abstriche wurden mit der Temperatur der letzten 7 Tage korreliert. Bei allen Bakterien sah man eine ähnliche Temperaturrange in der Vorwoche vor der Abstrichentnahme (Tab. 4).

**Table 4:** 

Keimspektrum	Parameter	Anzahl *n, *Häufigkeit	Mittelwert	Standardabweichung	50 % (Median)
Normalflora	Temperaturminimum (°C)	155	4,89	6,62	6,00
	Temperaturmaximum (°C)	155	22,08	8,52	24,00
	Temperaturrange, Durchschnitt (°C)	155	9,51	2,84	9,84
	Temperaturdurchschnitt (°C)	155	12,28	7,22	13,37
Streptokokken	Temperaturminimum (°C)	235	3,71	7,38	3,10
	Temperaturmaximum (°C)	235	19,99	8,97	21,00
	Temperaturrange, Durchschnitt (°C)	235	9,03	2,76	9,14
	Temperaturdurchschnitt (°C)	234	10,78	7,63	10,40
Staphylokokken	Temperaturminimum (°C)	39	3,54	6,38	3,80
	Temperaturmaximum (°C)	39	19,02	8,42	20,00
	Temperaturrange, Durchschnitt (°C)	39	8,69	2,77	9,09
	Temperaturdurchschnitt (°C)	39	10,36	6,77	10,07
Fusobacterium	Temperaturminimum (°C)	55	2,38	7,40	1,70
	Temperaturmaximum (°C)	55	19,32	9,04	18,00
	Temperaturrange, Durchschnitt (°C)	55	9,08	2,66	9,16
	Temperaturdurchschnitt (°C)	55	9,76	7,60	7,86
Mischinfektion (mehrere Keime nachweisbar)	Temperaturminimum (°C)	14	1,79	5,94	0,85
	Temperaturmaximum (°C)	14	19,57	7,06	17,20
	Temperaturrange, Durchschnitt (°C)	14	8,47	2,93	8,03
	Temperaturdurchschnitt (°C)	14	9,06	6,42	7,47

### Keimspektrum im Abstrich

Es waren 150/1450 Patienten (*n* = 57 Peritonsillitis und *n* = 193 PTA) ambulant bereits mit einem Antibiotikum vorbehandelt. Das häufigste orale Antibiotikum war Cefuroximaxetil mit 6,9 % (Tab. 5).

**Table 5:** 

Anbehandelt mit	Peritonsillitis: Anteil in % (Anzahl *n* der Patienten)	PTA: Anteil in % (Anzahl *n* der Patienten)	Gesamt: Anteil in % (Anzahl *n* der Patienten)
Keine orale antibiotische Therapie	78,9 (213)	83,6 (987)	82,8 (1200)
Cefuroxim	8,1 (22)	6,6 (78)	6,9 (100)
Penicillin	3,7 (10)	3,2 (38)	3,3 (48)
Amoxicillin	4,4 (12)	4,3 (51)	4,3 (63)
Clindamycin	0,7 (2)	0,4 (5)	0,5 (7)
Anderes	4,1 (11)	1,8 (21)	2,2 (32)

Bei 557/1180 (47,2 %) der PTA-Patienten erfolgte ein mikrobiologischer Abstrich. 12,9 % der Abstriche zeigten eine Normalflora, 19,7 % zeigten Streptokokken, 4,6 % Fusobakterien, 3,1 % Staphylokokken, mehrere Keime lagen in 1,2 % der Fälle vor. In 5,0 % der Fälle ergab sich der Nachweis seltener Bakterien. Patienten mit nicht auswertbaren Abstrichen wurden ausgeschlossen.

Die häufigste stationäre intravenöse antibiotische Therapie erfolgte mit Ampicillin/Sulbactam (48,7 %), gefolgt von Cefuroximaxetil (13,2 %) und Clindamycin (7,8 %).

### Entzündungswerte

Bei der Erstvorstellung lag das C‑reaktive Protein (CRP) bei allen Patienten im Mittel bei 8,96 mg/dl (Norm < 0,5 mg/dl), die Leukozyten im Mittel bei 13,74 G/l (Norm 4,0–9,0 G/l).

Bei den Peritonsillitiden lag das CRP im Mittel bei 7,82 mg/dl, bei PTA bei 9,23 mg/dl. Die Leukozyten lagen bei Peritonsillitiden im Mittel bei 12,76 G/l, beim PTA bei 13,97 G/l. Sowohl das CRP als auch die Leukozyten waren beim PTA signifikant höher (*p* = 0,009 für CRP; *p* < 0,001 für Leukozyten).

Die mittlere Krankenhausaufenthaltsdauer war 5,15 Tage (Minimum 1 Tag, *n* = 15 Patienten, und Maximum 42 Tage, *n* = 2 Patienten). Hierbei handelte es sich bei beiden Patienten um eine nekrotisierende Fasziitis als fulminante Komplikation eines initialen PTA.

### Klinische Symptomatik

Eine Kieferklemme zeigte sich bei 60/270 (22,2 %) der Peritonsillitiden und bei 716/1180 (60 %) der PTA. Es bestand eine signifikante Korrelation zwischen einer Kieferklemme und dem Vorhandensein eines PTA (*p* < 0,001). Das Risiko eines PTA war bei den Patienten, die eine Kieferklemme bei Erstvorstellung hatten, mehr als fünfmal so groß wie bei den Patienten mit einer Peritonsillitis (Odds Ratio [OR]: 5,40; 95%-KI: 3,96; 7,36).

### Rezidivierende Tonsillitiden

Es gaben 236/270 (87,4 %) Patienten mit einer Peritonsillitis und 1027/1180 (87,9 %) der Patienten mit einem PTA keine rezidivierenden Tonsillitiden in der Vergangenheit an.

Hierbei bestand kein statistisch signifikanter Unterschied zwischen Peritonsillitis/PTA für rezidivierende Tonsillitiden in der Vergangenheit (*p* = 0,86). Das mittlere Alter von Patienten mit einem PTA mit rezidivierenden Tonsillitiden lag bei 31,62 Jahren, ohne rezidivierende Tonsillitiden bei 36,88 Jahren. Patienten mit rezidivierenden Tonsillitiden waren signifikant jünger als Patienten ohne (*p* < 0,001).

### Operative Therapieoptionen

Die PTA traten bei 48 % rechts und 50,7 % links auf. Bei 15 Patienten (1,3 %) zeigte sich ein PTA beidseits. Bei 31,5 % der Patienten mit PTA erfolgte eine Spaltung des Abszesses, bei 68,5 % eine Operation. Hiervon wurden 20,8 % einseitig abszesstonsillektomiert und 48,2 % beidseitig.

**Figure 3:**
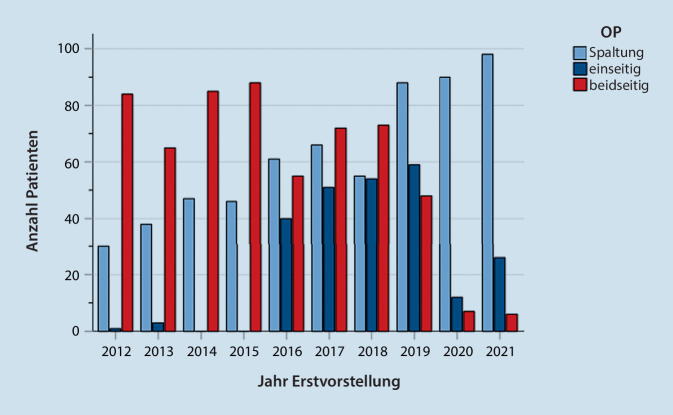

Im Verlauf von 10 Jahren zeigte sich ein deutlicher Trend von der beidseitigen zur einseitigen Abszesstonsillektomie und mittlerweile fast ausschließlich zur Spaltung unter Belassen der Tonsille. Nur auf Wunsch der Patienten, bei rezidivierenden Tonsillitiden oder Persistenz der Beschwerden erfolgte die Entscheidung zu einer Tonsillektomie (Abb. 3).

### Risiko einer Nachblutung

6,7 % der operierten Patienten zeigten eine Nachblutung. Hierbei ließ sich keine statistisch signifikante Korrelation zwischen der operierten Abszessseite und der Seite der Nachblutung nachweisen (*p* = 0,209). Im Kollektiv nahmen 5,2 % aller Patienten mit Acetylsalicylsäure (ASS) ein Präparat zur Thrombozytenaggregationshemmung ein. Von den Patienten, die eine Operation unter ASS erhielten, hatten 6,2 % eine postoperative Nachblutung. Es zeigte sich auch hier keine statistisch signifikante Abhängigkeit zwischen der Einnahme von ASS und einer postoperativen Nachblutung (*p* = 0,836). Von den Nachblutungen waren 23,75 % revisionsbedürftig, 76,25 % der Nachblutungen waren konservativ beherrschbar, u. a. mittels lokaler oder systemischer Therapie vasokonstriktorischer Medikamente und/oder bipolarer Elektrokoagulation in Lokalanästhesie.

### Komplikationen

Bei 114/1450 Patienten erfolgte eine Computertomographie. Die Indikation zu einer Schnittbildgebung erfolgte patientenindividuell u. a. bei Verdacht auf eine fortschreitende Abszedierung über den Peritonsillarraum hinaus oder Schwierigkeiten in der Diagnosefindung, z. B. bei einer ausgeprägten Kieferklemme und/oder schwieriger Beurteilung der lokalen Ausdehnung vor allem Richtung Hypopharynx oder einer Punctio sicca. Aber auch die Expertise des Arztes vor allem im Dienst spielten hierbei eine Rolle. Bei 56 Patienten (49,14 %) zeigte sich hierbei ein „normaler“ PTA, bei 12 Patienten (10,53 %) ein Retrotonsillarabszess, bei 39 Patienten (34,21 %) ein Parapharyngealabszess, bei drei Patienten ein dentogener Fokus, bei einem Patienten eine nekrotisierende Fasziitis und bei drei Patienten kein bildmorphologischer Abszess.

## Diskussion

Ziel dieser retrospektiven Studie war die Analyse der Häufigkeit von PTA in Abhängigkeit von Temperaturschwankungen. Eingeschlossen wurden 1450 Patienten, die zwischen 2012 und 2021 (10 Jahre) mit einer Peritonsillitis/PTA in der Klinik für Hals‑, Nasen‑, Ohrenheilkunde des Klinikums rechts der Isar der Technischen Universität München stationär behandelt wurden. In Übereinstimmung mit der Literatur lag der mittlere Altersdurchschnitt in unserem Kollektiv bei 36,21 (± 15,97) Jahren [3, 11, 22].

Es zeigte sich keine Korrelation zwischen der Häufigkeit von Abszessen und der Temperatur von Beschwerdebeginn bis zum Abszess. Auch bei maximalen Temperaturschwankungen zwischen heiß und kalt (Range) kam es zu keiner statistisch relevanten Häufung. Über das ganze Jahr verteilt sah man eine ähnliche Inzidenz von Peritonsillitiden/PTA. Diese Ergebnisse stimmen mit der Literatur überein [16, 18, 22]. Jedoch wurden hierbei die Daten nie patientenbezogen auf die Temperatur von Beginn der Symptome bis zum Auftreten des PTA korreliert, was das Besondere in dieser Studie war. Ein kalter Sommer bzw. warmer Winter mit stärkeren Temperaturschwankungen wurde nie berücksichtigt. In einigen anderen Studien wurde hingegen eine unterschiedliche Inzidenz von PTA über das Jahr verteilt festgestellt [24, 29, 31].

Der PTA wird oft als Komplikation einer fortschreitenden Infektion der oberen Atemwege angesehen, die mit einer akuten Pharyngitis bzw. Tonsillitis beginnt. Einige Studien haben das vermehrte Auftreten von Tonsillitiden in den Wintermonaten beschrieben [17, 18]. Ebenso traten virale Infektionen der oberen Atemwege, ausgelöst u. a. durch Rhinoviren, Adenoviren, Metapneumoviren, Coronaviren, sowie akute Bronchitiden vermehrt bei kalten Umgebungstemperaturen auf [6, 30]. Unsere Studie zeigte jedoch, dass weder die Temperaturhöhe noch die Range einen Einfluss auf die Häufigkeit von Peritonsillitiden und PTA hat. Sie traten über das Jahr mit ähnlicher Inzidenz auf, was die Theorie der Entwicklung des Peritonsillarabszesses aus einer akuten Tonsillitis vor allem in den Wintermonaten infrage stellt. In einer weiteren Studie konnte auch keine Korrelation zwischen der Umgebungstemperatur und der Häufigkeit des Auftretens von dentalen Abszessen festgestellt werden [28].

Bei 47,2 % der Patienten mit PTA erfolgte die Durchführung eines mikrobiologischen Abstrichs. Die Entscheidung zur Abstrichentnahme erfolgte individuell. Ein Abstrich erfolgte bei ausgeprägten Abszessen, hohen laborchemischen Infektparametern oder einem komplikativen Verlauf. Auf einen Abstrich wurde oft bei einem reinen PTA und auch u. a. am Wochenende, aufgrund des Risikos einer Verfälschung der Ergebnisse durch die Lagerung bis zum Wochenbeginn, verzichtet. Bei 12,9 % der Abstriche zeigte sich eine Normalflora. Auch die hohe Rate an apathogenen Erregern in den Ergebnissen führte zu einer Reduktion der Abstrichentnahmen.

Die häufigsten diagnostizierten Erreger waren in 19,7 % Streptokokken der Gruppe A (GAS). Da die Tonsillen gesunder Personen bereits häufig mit Bakterien kolonisiert sind, stellt die Identifizierung pathogener Erreger zum Teil eine Herausforderung dar. Die Entnahme der Abstriche, die Aufbewahrung, der Transport und eine bereits eingeleitete Antibiotikabehandlung können die Diagnostik beeinflussen [16].

Unsere Ergebnisse bestärken die Empfehlung der S2k-Leitlinie „Antibiotikatherapie bei HNO-Infektionen“, dass eine mikrobiologische Diagnostik meist nicht notwendig ist. Des Weiteren liegen die Abstrichergebnisse erst nach Beginn der intravenösen antibiotischen Therapie vor und haben meist keine Konsequenz beim immunkompetenten Patienten. Lediglich bei fulminanten Verläufen mit Persistenz und Verschlechterung bis hin zur nekrotisierenden Fasziitis oder einer Mediastinitis trotz genannter Therapie ist die angepasste antibiotische Therapie nach Antibiogramm ratsam.

Es gibt bisher keine Studien, die das Erregerspektrum intraoperativer Abstriche in Abhängigkeit von der Außentemperatur und der Temperaturrange individuell untersuchten. Sowohl beim Nachweis der jeweiligen Erreger (Streptokokken, Staphylokokken, Fusobacterium und Mischinfektionen) als auch beim Nachweis einer Normalflora im Abstrich zeigte sich eine ähnliche Temperaturrange. Es zeigte sich keine Korrelation zwischen der Häufigkeit des Auftretens von bestimmten Erregern und der Temperatur in der Woche vom Symptombeginn bis zum Auftreten des PTA. Klug et al. stellten in einer Studie fest, dass Fusobakterien und GAS vermehrt in den Sommermonaten auftraten [16], führten jedoch keine Untersuchung zur Temperaturschwankung durch. Unsere Ergebnisse unterstützen die Theorie, dass PTA nicht nur eine direkte Komplikation einer akuten Tonsillitis darstellen, da einige Studien bereits feststellten, dass die Inzidenz von GAS-positiven akuten Tonsillitiden eine deutliche saisonale Variation zeigte [2, 13, 18]. Es ist eine mögliche Komplikation der Peritonsillitis, kann jedoch nicht als alleiniger Faktor gelten.

International herrscht kein Konsens über die empfohlene intravenöse antibiotische Therapie von Peritonsillitiden/PTA [15, 33, 34]. Die am häufigsten durchgeführte intravenöse antibiotische Therapie erfolgte mit Ampicillin/Sulbactam, entsprechend der S2k-Leitlinie [9]. Das häufigste orale Antibiotikum vor Erstvorstellung war Cefuroximaxetil (8,1 % bei Peritonsillitis und 6,6 % bei PTA). 78,9 % der Patienten waren nicht anbehandelt. Die orale Bioverfügbarkeit von Cefuroximaxetil ist stark eingeschränkt. Verbessert wird die Bioverfügbarkeit durch Einnahme mit einer Mahlzeit, jedoch liegt die orale Bioverfügbarkeit von Cefuroximaxetil auch in der optimalen Situation nur bei 50 %. Dies verdeutlicht, dass orale Breitspektrumantibiotika mit eingeschränkter Bioverfügbarkeit vermieden werden sollten [8, 9]. Ob Cefuroxim häufiger gegeben wurde und deshalb am häufigsten genannt wurde oder ob es wirklich an der eingeschränkten Wirksamkeit liegt, was das Auftreten des Abszesses nicht verhinderte, lässt sich in diesem Kontext nicht eruieren. Dennoch zeigt es deutlich, dass die Entscheidung zu einem potenteren Antibiotikum wie Amoxicillin/Clavulansäure bzw. Ampicillin/Sulbactam ratsam ist.

Symptome von Patienten mit einer Peritonsillitis/PTA sind einseitige Odynophagie und Schluckunfähigkeit. Eine Kieferklemme zeigte sich bei nur 22,2 % der Peritonsillitiden, jedoch bei 60,0 % der PTA. Das Risiko eines PTA bei Patienten mit Kieferklemme war mehr als fünfmal so groß, im Vergleich zu Patienten mit einer Peritonsillitis. Die Kieferklemme kann im ambulanten hausärztlichen Setting als ein wichtiges Entscheidungskriterium für eine stationäre Einweisung dienen.

Therapie der Wahl beim PTA ist die Drainage des Abszesses sowie eine antibiotische Therapie. International geht der Trend zu weniger invasiven chirurgischen Ansätzen mittels Abszessspaltung in Lokalanästhesie und Vermeidung einer Tonsillektomie à chaud [16, 25, 32]. Dies zeigte sich auch in unserem Kollektiv mit einer deutlichen Regredienz der Tonsillektomie und einer Zunahme der Spaltung, bedingt durch die Anpassung der Leitlinien und/oder der Präferenz der behandelnden Ärzte. Argumente für die Spaltung sind die geringe Invasivität, kürzere Krankenhausaufenthaltsdauer, geringere Nachblutungsrate sowie die geringe Rate von 12 % an Patienten mit rezidivierenden akuten Tonsillitiden in der Vorgeschichte. In der Literatur schwanken die Daten zur Häufigkeit von rezidivierenden Tonsilliden in der Vorgeschichte vor PTA stark (zwischen 0 und 56 %) [19, 22–25, 29, 32, 36, 37]. In unserem Kollektiv zeigten nur drei Patienten ein Rezidiv eines PTA. Wenn man davon ausgeht, dass Patienten sich mit einem Rezidiv in der gleichen Klinik vorstellen, erwies sich die im Vergleich zur Abszesstonsillektomie weniger invasive Inzisionsdrainage als ausreichend. Eine Abszesstonsillektomie ist zu bevorzugen bei Komplikationen durch den Peritonsillarabszess oder bei Persistenz trotz konservativer Therapieverfahren [3]. Die bevorzugte chirurgische Behandlung hängt von den Patienten, der Compliance und der Erfahrung des konsultierten Arztes ab. Auch in der Literatur zeigten mehrere Studien gute Wirksamkeitsraten mittels Spaltung im Vergleich zur Tonsillektomie [11, 14, 25].

Die Rezidivraten schwanken in der Literatur zwischen 9,8 und 13,9 % [7, 12]. Als Risikofaktoren für ein Rezidiv wurden hierbei rezidivierende Tonsillitiden in der Vorgeschichte sowie eine bildmorphologische Abszessausdehnung über das peritonsilläre Gewebe hinweg festgestellt [7]. Dies verdeutlicht, dass hauptsächlich bei Patienten mit rezidivierenden PTA bzw. rezidivierenden Tonsillitiden in der Vorgeschichte, mit einer Ausbreitung des Abszesses über das peritonsilläre Gewebe hinweg, frustraner Therapie mittels Inzisionsdrainage bzw. Abszesspunktion, oder einer fehlenden Compliance des Patienten eine einseitige/beidseitige Tonsillektomie in Erwägung gezogen werden sollte.

Trotz der größten Bemühungen aller Chirurgen, sie zu vermeiden, bleibt die Nachblutung die bedeutendste, manchmal lebensbedrohliche Komplikation bei Tonsillektomien. Die Nachblutungsrate von lediglich 6,7 % entspricht in etwa der durchschnittlichen Nachblutungsrate nach Tonsillektomie in Deutschland, welche nach Auswertungen von ca. 1500 Patienten zwischen 2005 und 2017 bei Frauen bei 5,41 % und bei Männern bei 7,64 % lag [35], jedoch unter der in der Literatur beschriebenen durchschnittlichen Nachblutungsrate nach Abszess-Tonsillektomien [7, 12] Im Hinblick auf das Nachblutungsrisiko von 6,7 % konnte keine signifikante Korrelation zwischen der Abszessseite und der Seite der Nachblutung nachgewiesen werden. Bei den erhobenen Daten handelt es sich um Nachblutungen, die während des stationären Aufenthalts oder nachstationär auftraten. Es wurden nur die Nachblutungen der Patienten mit Zustand nach Abszess-Tonsillektomie eingeschlossen. Hierbei wurde von einer Wiedervorstellung bei uns als operierender Klinik ausgegangen.

## Limitationen

Diese Studie hat einen retrospektiven Ansatz. Es konnten nur die Daten berücksichtigt werden, die zum Behandlungszeitraum vorlagen. In Bezug auf das Nachblutungsrisiko wurden die Daten, die während des stationären Aufenthalts oder nachstationär auftraten, ausgewertet. Es wurde dabei von einer Wiedervorstellung in der operierenden Klinik ausgegangen, jedoch kann eine Vorstellung in einem anderen Krankenhaus bei einer Nachblutung nicht vollständig ausgeschlossen werden.

In der vorliegenden Studie wurde der Diagnoseschlüssel J36.0 nach ICD 10 für Peritonsillitis/PTA ausgewertet und anhand der digitalen Krankenakten die Informationen über klinische Symptome, Beschwerdedauer, Alter, Einweisungsdiagnose, Aufenthaltsdauer, Therapie und Infektparameter. Es kann daher nicht sicher ausgeschlossen werden, dass sich Patienten nach einer Therapie des PTA mittels Inzisionsdrainage nach einer gewissen Zeit einer elektiven TE unterzogen haben. Dies war jedoch auch nicht die Fragestellung in dieser Auswertung.

## Fazit für die Praxis

- In diese Studie wurden 1450 Patienten eingeschlossen, welche zwischen Januar 2012 und Dezember 2021 mit einer Peritonsillitis oder einem Peritonsillarabszess in der Klinik und Poliklinik für Hals‑, Nasen‑, Ohrenheilkunde des Klinikums rechts der Isar der Technischen Universität München behandelt wurden.
- Es zeigte sich über das ganze Jahr eine ähnliche Häufigkeit im Auftreten von Peritonsillitiden und Peritonsillarabszessen.
- Es zeigte sich keine Korrelation zwischen dem Auftreten von Peritonsillarabszessen und der Umgebungstemperatur, auch zeigte sich insbesondere keine Korrelation zwischen einer hohen Temperaturrange und der Häufigkeit des Auftretens von Abszessen
- Der Mythos eines Abszesswetters bei Peritonsillarabszessen konnte somit widerlegt werden.
- Eine Herausforderung vor allem im ambulanten hausärztlichen Setting stellt häufig die Differenzierung zwischen einer Peritonsillitis und dem Peritonsillarabszess dar.
- Unsere Auswertung bestätigte die Kieferklemme als eines der wichtigsten Kriterien zur Entscheidung für eine stationäre Einweisung.
- Auch bestätigte sich, dass die orale Gabe von Cefuroximaxetil aufgrund einer Bioverfügbarkeit von nur ca. 50 % vermieden werden sollte.
- Ob Cefuroxim in den letzten 10 Jahren insgesamt häufiger gegeben wurde als andere orale Antibiotika und deshalb am häufigsten genannt wurde oder ob es wirklich an der eingeschränkten Wirksamkeit liegt, lässt sich in diesem Kontext nicht eruieren, dennoch zeigt unsere Untersuchung deutlich, dass die Entscheidung zu einem Antibiotikum mit höherer oraler Bioverfügbarkeit ratsam ist.
